# Changes in hair cortisol and oxytocin independently associate with positive and negative psychological states in female soccer players

**DOI:** 10.3389/fspor.2025.1742869

**Published:** 2026-01-12

**Authors:** Nodoka Ohara, Hana Kameo, Genta Ochi

**Affiliations:** 1 Department of Health and Sports, Niigata University of Health and Welfare, Niigata, Japan; 2 Institute for Human Movement and Medical Sciences, Niigata University of Health and Welfare, Niigata, Japan

**Keywords:** chronic fatigue, conditioning, female athlete, hair hormone level, over-training

## Abstract

**Introduction:**

This study examined changes in hair cortisol concentration (HCC) and hair oxytocin concentration (HOC) in female athletes and aimed to clarify their relationships with training load, daily life stressors, and mental health indicators.

**Methods:**

Hair samples were collected from 22 female university soccer players (age: 19.8 ± 0.8 years), and psychological assessments were conducted in February and March 2024. Cortisol and oxytocin were extracted and measured from hair, and their associations with mood states [measured using the Profile of Mood States 2nd Edition (POMS2)], psychological distress [measured using the Kessler Psychological Distress Scale-6 (K6) scale], and self-reported training load were analyzed.

**Results:**

Training load and POMS fatigue levels increased significantly from February to March (t = 4.27, *P* < 0.001 and t = 4.74, *P* < 0.001, respectively); however, despite these increases in physical demands and perceived fatigue, no significant changes were observed in HCC and HOC. Regression analysis using M-estimation revealed that changes in academic stress were significantly negatively associated with HCC changes (*β* = −1.04, *p* = 0.026), and changes in training load showed a positive association trend with HOC changes (*β* = 0.0003, *p* = 0.068). Spearman correlation analysis showed a significant negative correlation between HCC changes and POMS vigor changes (rs = −0.44, *p* = 0.039) and a significant positive correlation between HOC changes and K6 scale changes (rs = 0.43, *p* = 0.043). No significant correlation was found between HCC and HOC.

**Discussion:**

HCC and HOC fluctuated independently and were associated with distinct psychological outcomes: HCC changes correlated with vigor, while HOC changes correlated with psychological distress. These findings suggest that each hormone may capture different dimensions of chronic stress and psychological functioning.

**Conclusion:**

Combined measurement of HCC and HOC may be useful in assessing chronic stress in female soccer players, providing complementary information for athlete health management.

## 1 Introduction

Athletes engaging in intense daily training to achieve high performance may be in a constant state of considerable stress. Insufficient rest, nutrition, or excessive training can cause severe stress, potentially leading to overtraining syndrome, characterized by decreased performance and mood (1, 2). Furthermore, increased risk-taking behaviors due to chronic mental and physical stress may cause injuries (3). Understanding stress states may help prevent poor performance, decreased mental health, burnout, and dropout. Studies in sport psychology and athlete monitoring research have employed questionnaire-based methods to assess stress states; however, psychological indicators can be influenced by events at the time of measurement (e.g., diet or training), allowing temporary mood to affect the results (4). Additionally, because the results of mental health and stress measurements may influence evaluations by coaches or managers, athletes may exhibit social desirability bias and underreport their symptoms (5). Therefore, physiological assessment methods for chronic stress that are not influenced by temporary mood at the time of measurement should be developed.

Salivary cortisol has been widely used as an acute stress biomarker in sport science research, providing real-time assessment of hypothalamic-pituitary-adrenal (HPA) axis activity (6–8). However, salivary measurements reflect transient hormonal fluctuations and may be influenced by circadian rhythms, recent meals, and acute stressors at the time of collection. In contrast, hair-based hormone measurements offer an opportunity to assess cumulative hormone exposure over extended periods. Recent research has suggested that hormones accumulated in human hair can serve as useful physiological indicators of long-term stress (9). Hair grows approximately 1 cm per month (10), and blood-derived hormones are assumed to accumulate via capillaries during hair formation (11). Cortisol, commonly used as a stress indicator, accumulates in hair, with elevated levels reported in relation to stressful life events (12, 13) and during pregnancy (14). Hair cortisol concentration (HCC) has been found to correlate more strongly with physical stressors, such as intense physical activity, than with psychological stress (15), leading to higher HCC values in individuals with high physical activity levels and athletes who engage in rigorous exercise (16). This relationship reflects not only inter-individual differences but also intra-individual variations, as increases in training load among female athletes have been associated with elevated HCC (17). Notably, questions remain regarding sex-based differences in HCC (9, 18), and female athletes may exhibit distinct stress reactivity patterns due to fluctuations in ovarian hormones across the menstrual cycle, which can interact with HPA axis activity. Furthermore, the impact of HCC on mental health has been reported to be stronger in women than in men (19), suggesting that female athletes may be particularly susceptible to the psychological effects of chronic cortisol elevation. However, whether increased chronic stress is associated with a decline in mental health remains unclear. Previous research on hair cortisol has suggested that elevated HCC in athletes may contribute to low mood and reduced aerobic endurance performance (20); yet, this association with mood decline was not replicated in female athletes. Endocrine responses to stress reactions involve not only cortisol but multiple hormones. Therefore, the degree of increase in HCC and its effects on mood and performance may be regulated by other hormones that influence individual and group differences.

Oxytocin is a peptide hormone associated with social cognition and attachment (21) and is secreted during novel events (22, 23) and stress responses (24). Acute oxytocin administration has been shown to modulate stress responses by binding to hypothalamic receptors and exerting inhibitory effects on the hypothalamic-pituitary-adrenal (HPA) axis (25–27), attenuating stress responses (28) and exerting anxiolytic effects (29). However, recent animal studies have reported a positive correlation between hair oxytocin and cortisol (30), suggesting that both hormones may increase together under chronic stress conditions rather than oxytocin effectively suppressing cortisol accumulation. It should be noted that this evidence comes primarily from animal research, which may limit its generalizability to human populations. Nevertheless, this finding raises the possibility that elevated hair oxytocin concentration (HOC) may serve as a marker of chronic stress exposure rather than an indicator of successful stress regulation. While many studies have examined the transient effects of oxytocin on stress responses, the relationship between chronic oxytocin levels and long-term stress outcomes remains unclear. Similar to cortisol, the amount of oxytocin accumulated in hair can be measured using similar extraction methods. If individuals with higher HOC levels also show elevated hair cortisol and impaired mood or performance, this would suggest that chronic stress exposure increases both hormones simultaneously. This pattern could reflect a compensatory but insufficient stress response mechanism, wherein oxytocin is released in an attempt to buffer stress but fails to adequately downregulate HPA axis activity under conditions of chronic load (25, 26).

Therefore, this study aimed to determine whether oxytocin in hair modulates cortisol secretion and mood effects in female athletes using a two-month longitudinal study with 22 female soccer players. We hypothesized that: (1) increases in training load would be associated with increases in HCC and/or HOC, and (2) changes in these hair hormones would be associated with changes in mood states and psychological distress.

## 2 Methods

### 2.1 Participants

Twenty-two members of the women's soccer club of Niigata University of Health and Welfare consented to participate in this study during the 2024 season (age 19.8 ± 0.8 years, height 160.3 ± 5.5 cm, weight 54 ± 4.7 kg, soccer experience 13.8 ± 1.7 years). Playing positions included goalkeepers (*n* = 3), defenders (*n* = 7), midfielders (*n* = 10), and forwards (*n* = 2). Hair color was not systematically recorded; however, all participants were Japanese females with naturally black or dark brown hair, minimizing potential confounding effects of hair pigmentation on hormone extraction efficiency (31). For body composition, the players were asked to complete a questionnaire based on the results of their physical examinations received in January.

We informed participants about the purpose, content, and safety of the study and obtained their written consent to participate in the study. The study was conducted in accordance with the principles of the Declaration of Helsinki. The Ethics Committee of the Niigata University of Health and Welfare approved this study (approval number: 18881-220831).

The *post-hoc* power analysis revealed that with our sample size of 22 participants, a statistical power of 0.80, and a significance level of 0.05 (two-sided), the minimum detectable effect size (rs) was 0.559.

### 2.2 Experimental procedures

Hair samples were collected, and psychological questionnaires were administered in the second weeks of February and March. January was the off period during the 2024 season, and team training began in February. The day before the measurement, the participants were given a day off from club activities and were asked to report to the laboratory between 10:00 and 14:00. They were asked to refrain from eating or drinking for 2 h before the meeting to standardize conditions for psychological assessments. During the measurement period, all participants were asked to participate in the activities of their football club. The raters took only two measurements and provided no instructions for any other activity.

### 2.3 HCC and HOC

Hair samples were collected from the back of the head with minimal variation (32). To assess chronic stress levels during the previous month, we chose a 1-cm section (10 mg) from the scalp end of the collected hair for analysis, drawing on previous studies (17, 33). Hair samples were collected using scissors and weighed using an electronic balance (HT84R; Shinko Denshi, Japan). As human hair grows approximately 1 cm per month (10, 31, 32), it was assumed that the hair samples from the February and March measurement periods would be completely different. The hair was washed twice with isopropanol for 3 min at room temperature to remove any sweat or sebum secretions adhering to the hair surface, following established and validated protocols (17, 20). The washed hair was then air-dried and weighed. Washed hair samples were outsourced to Air Plants Bio Co., Ltd. (Japan) for hormone extraction and quantification using enzyme immunoassay. Standard curves demonstrated excellent fit (*r*^2^ = 0.999 for cortisol; *r*^2^ = 0.998 for oxytocin), with detection ranges of 0.0488–50 ng/mL for cortisol and 7.8125–2,000 pg/mL for oxytocin. Sample coefficients of variation were generally below 10%. The measurement methods were identical to those used in previous studies (17).

### 2.4 Psychological measurements

The participants completed the Profile of Mood States 2nd Edition (POMS2), the Kessler Psychological Distress Scale-6 (K6) scale, and a self-reported training load.

### 2.5 POMS2

The POMS2 (34) comprises 35 items that assess seven mood states (anger–hostility, confusion–bewilderment, depression–ejection, fatigue–inertia, tension–anxiety, vigor–activity, and friendliness). In this study, we used only the ten-item subsets of vigor–activity and fatigue–inertia to minimize the participants’ psychological burden. The internal consistency reliability (Cronbach's alpha) of this scale in this study was 0.86 (vigor in February), 0.92 (vigor in March), 0.56 (fatigue in February), and 0.59 (fatigue in March). The relatively low internal consistency for the fatigue subscale (0.56–0.59) should be noted as a limitation when interpreting fatigue-related findings.

### 2.6 K6 scale

We used the Japanese version of the K6 scale, which is a powerful screening tool for psychological distress (35). Respondents rated six items on a five-point Likert scale, with scores ranging from 0 to 4. A higher total score indicated poor mental health. Moderate or greater psychological distress was defined as a K6 scale score of ≥5 (36, 37). The internal consistency reliability (Cronbach's alpha) of the scale in this study was 0.79 (February) and 0.92 (March).

### 2.7 Self-reported training load

We calculated the self-reported training load as the product of monthly training hours and intensity. We used an 11-point scale ranging from 0 to 10 to measure the rate of perceived exertion (38). Self-reported training load, assessed monthly in retrospect, has been validated as having a strong correlation with training volume measured using a heart rate monitor (4). The internal consistency reliability (Cronbach's alpha) of the scale in this study was 0.37 (February) and 0.57 (March).

### 2.8 Daily life stressor scale for university students

The measurement of daily life stressors was conducted using the shortened version of Shima's (39) Daily Life Stressor Scale for University Students, comprising 24 items across four subscales: existential (self) stressors related to identity and life direction (6 items), interpersonal stressors related to social relationships (8 items), academic stressors related to university life and coursework (6 items), and physical stressors related to health and bodily concerns (4 items). The academic stressor subscale includes items assessing concerns such as uninteresting classes, unsatisfactory grades, difficulty of exam preparation, difficulty keeping up with classes, anxiety about advancing to the next grade, and difficulty preparing reports and seminars. The physical stressor subscale assesses daily-life physical concerns (e.g., poor physical condition, physical fatigue, injury or illness), which are conceptually distinct from training load assessed separately in this study. Using a five-point Likert-type response format (0 = did not experience, 1 = hardly bothered, 2 = slightly bothered, 3 = considerably bothered, 4 = extremely bothered), participants indicated whether they experienced each stressor during the past three months and, if so, how much it bothered them. Shima (1992) reported that this scale exhibited satisfactory convergent validity when compared with Goldberg's (1978) General Health Questionnaire, yielding correlation coefficients between 0.48 and 0.61.

### 2.9 Statistical analyses

All statistical analyses were performed using R version 4.4.2 (R Core Team, Vienna, Austria). Descriptive statistics (mean, standard deviation, median, and range) were calculated for all variables at both time points (February and March 2024). Normality of the data was assessed using the Shapiro–Wilk test. Changes between the two time points were examined using paired t-tests for normally distributed variables, while Wilcoxon signed-rank tests were used for non-normally distributed variables. Statistical significance was set at *p* < 0.05. To examine the relationships between changes in stress factors and changes in stress hormones, robust regression analyses with M-estimation (Huber method) were conducted using the `rlm` function from the MASS package. This approach was chosen because the HCC change scores showed non-normal distribution (Shapiro–Wilk test: W = 0.82, *p* = 0.001), and regression with M-estimation (Huber method) is more resistant to outliers and violations of normality assumptions compared to ordinary least squares regression. Two separate models were constructed: (1) HCC change as the dependent variable and (2) HOC change as the dependent variable. For both models, changes in self-reported training load and four stress factors (existential, interpersonal, academic, and physical aspects) served as independent variables. Multicollinearity was assessed using variance inflation factors (VIF), with VIF < 10 considered acceptable. Model fit was evaluated using pseudo-*R*^2^ values. Relationships between changes in stress hormones and changes in mental health indices [K6 scale, Fatigue–inertia (POMS2), and Vigor–activity (POMS2)] were examined using Spearman's rank correlation coefficients, given the non-parametric nature of some variables. Statistical significance was set at *p* < 0.05.

## 3 Results

### 3.1 Descriptive statistics and normality

Normality tests revealed that cortisol change scores (W = 0.82, *p* = 0.001) and K6 scale change scores (W = 0.90, *p* = 0.025) were non-normally distributed, while all other change scores showed normal distributions (*p* > 0.05).

### 3.2 Changes between measurement time points

Table 1 shows the results of paired comparisons between February and March. Training load significantly increased from February to March (t = 4.27, *p* < 0.001, Cohen's d = 0.91). POMS fatigue also showed a significant increase (t = 4.74, *p* < 0.001, Cohen's d = 1.01). Both effect sizes indicate large effects. POMS fatigue also showed a significant increase (t = 4.74, *p* < 0.001). No significant changes were observed for cortisol, oxytocin, K6 scale, POMS vigor, or any of the four stress factor categories.

**Table 1:** Changes in main variables between February and march measurements.

Variable	February mean ± SD	March mean ± SD	Mean change ± SD	Test method	Statistic	*p*-value	Significance
HCC (pg/mg)	10.27 ± 5.69	9.76 ± 3.07	−0.51 ± 6.20	Wilcoxon signed-rank	138.00	0.726	n.s.
HOC (pg/mg)	3.57 ± 1.03	3.83 ± 1.30	+0.26 ± 1.38	Paired *t*-test	0.89	0.382	n.s.
K6 scale (point)	8.64 ± 3.36	10.23 ± 4.53	+1.59 ± 4.82	Wilcoxon signed-rank	87.50	0.124	n.s.
Fatigue–inertia (POMS2; point)	2.36 ± 1.79	4.59 ± 2.58	+2.23 ± 2.20	Paired *t*-test	4.74	<0.001	***
Vigor–activity (POMS2; point)	6.41 ± 3.98	6.95 ± 3.99	+0.55 ± 4.24	Paired *t*-test	0.60	0.553	n.s.
Self-reported training load (AU)	1,384.09 ± 1,039.04	3,466.82 ± 1,788.12	+2,082.73 ± 2,288.74	Paired *t*-test	4.27	<0.001	***
Interpersonal stressor (point)	4.09 ± 3.87	5.45 ± 5.47	+1.36 ± 4.56	Paired *t*-test	1.40	0.176	n.s.
Physical aspects stressor (point)	5.14 ± 2.19	5.82 ± 2.95	+0.68 ± 3.08	Paired *t*-test	1.04	0.310	n.s.
Existential stressor (point)	7.45 ± 3.96	8.32 ± 5.00	+0.86 ± 3.03	Paired *t*-test	1.34	0.195	n.s.
Academic stressor (point)	5.64 ± 2.42	4.86 ± 3.01	−0.77 ± 2.74	Paired *t*-test	−1.32	0.200	n.s.

HCC, hair cortisol concentration; HOC, hair oxytocin concentration; n.s., not significant; ***p < 0.001.

### 3.3 Robust regression analyses predicting stress hormone changes

Two robust regression models were constructed to examine whether changes in training load and stress factors predicted changes in cortisol and oxytocin. VIF values for all predictors were below 2.0 in both models, indicating no problematic multicollinearity.

The cortisol model (Table 2) showed a significant negative association with academic stressor change (*β* = −1.04, *p* = 0.026), indicating that greater increases in academic stress were associated with smaller increases (or greater decreases) in cortisol levels. A positive association trend was found with physical aspects stressor change (*β* = 0.78, *p* = 0.062). The model explained 49.0% of the variance (pseudo-*R*^2^ = 0.490).

**Table 2:** Robust regression analysis predicting cortisol change.

Predictor	*β*	SE	t	*p*	Pseudo-*R*^2^
(Intercept)	−1.353	1.442	−0.94	0.362	0.490
Self-reported training load change	−0.000	0.000	−0.60	0.556	
Interpersonal stressor change	0.570	0.338	1.69	0.111	
Physical aspects stressor change	0.782	0.390	2.01	0.062	
Existential stressor change	−0.493	0.510	−0.97	0.348	
Academic stressor change	−1.043	0.424	−2.46	0.026*	

Robust regression with M-estimation (Huber method). *p < 0.05.

The oxytocin model (Table 3) showed a positive association trend with self-reported training load change (*β* = 0.0003, *p* = 0.068). Although this association did not reach the conventional threshold for statistical significance (*p* < 0.05), it suggests a potential relationship between training load increases and oxytocin accumulation that warrants further investigation with larger samples. No other predictors reached statistical significance. The model explained 29.6% of the variance (pseudo-*R*^2^ = 0.296).

**Table 3:** Robust regression analysis predicting oxytocin change.

Predictor	β	SE	t	*p*	Pseudo-*R*^2^
(Intercept)	−0.237	0.427	−0.55	0.587	0.296
Self-reported training load change	0.000	0.000	1.96	0.068	
Interpersonal stressor change	0.132	0.100	1.32	0.205	
Physical aspects stressor change	−0.062	0.115	−0.54	0.598	
Existential stressor change	−0.064	0.151	−0.43	0.676	
Academic stressor change	0.130	0.126	1.04	0.315	

Robust regression with M-estimation (Huber method).

### 3.4 Spearman correlations between stress hormone changes and mental health changes

Significant negative correlation was found between HCC change and Vigor–activity (POMS2)(rs = −0.44, *p* = 0.039), indicating that decreases in HCC were associated with increases in vigor-activity. Significant positive correlation was found between HOC change and K6 scale change (rs = 0.43, *p* = 0.043), suggesting that increases in HOC were associated with increases in psychological distress. No other correlations reached statistical significance. The results are shown in Table 4.

**Table 4:** Spearman correlations between stress hormone changes and mental health changes.

Hormone change	Mental health change	rs	*p*	*n*
HCC	K6 scale	−0.030	0.895	22
HCC	Fatigue–inertia (POMS2)	0.196	0.382	22
HCC	Vigor–activity (POMS2)	−0.443	0.039*	22
HOC	K6 scale	0.434	0.043*	22
HOC	Fatigue–inertia (POMS2)	0.251	0.260	22
HOC	Vigor–activity (POMS2)	−0.026	0.908	22

HCC, hair cortisol concentration; HOC, hair oxytocin concentration; rs, Spearman's rank correlation coefficient. *p < 0.05.

Regarding the relationship between the two stress hormones, no significant correlations were found between HCC and HOC at February (rs = −0.03, *p* = 0.900), March (rs = −0.24, *p* = 0.283), or in their changes (rs = 0.28, *p* = 0.213), indicating that these hormones responded independently to stress. The absence of significant correlations between HCC and HOC at any time point or in their changes is noteworthy, as it indicates that these two stress-related hormones respond independently to environmental demands in this population.

## 4 Discussion

In this study, we measured HCC and HOC at two time points, February and March, to evaluate chronic stress states in female soccer players and examined their relationships with training load, mood indicators, and daily life stressors. Training load and POMS fatigue levels increased significantly; however, no significant changes were observed in HCC and HOC.

Interestingly, although a previous study (17) found that increased training load was associated with elevated HCC, this study observed no significant changes in HCC between the two time points. One possible explanation for this discrepancy may be the seasonal differences in measurement timing, although this interpretation remains tentative and requires further investigation. A systematic review reported that most studies found lower HCC in winter/spring than in summer/autumn, showing a seasonal pattern (40). The previous study collected data between August and October, reflecting the period of intensive training and competitions in summer endurance athletes (17), whereas the present study conducted measurements in February and March, during the winter-to-spring transition. Winter/spring represents the nadir period for HCC (40), suggesting that HCC in the present study may have been influenced by seasonal reduction. Therefore, HCC may not have sensitively reflected the stress associated with increased training load during this measurement period, though this hypothesis awaits confirmation in future studies with year-round measurements.

Additionally, the robust regression analysis revealed that increases in academic stress were significantly associated with decreases (or attenuated increases) in HCC (*β* = −1.04, *p* = 0.026). This unexpected negative association requires careful interpretation. Several possibilities may explain this finding: (1) academic stress and training stress may have different temporal dynamics, with academic stress potentially reflecting anticipatory or cognitive strain rather than physical exertion that activates the HPA axis; (2) students experiencing higher academic stress during examination periods may reduce their training intensity or attendance, thereby leading to lower cortisol accumulation; or (3) this association may represent a statistical artifact due to the modest sample size and multiple comparisons. The effects of psychological vs. physical stress on hair hormones require detailed analysis in future research with larger samples.

Among the parameters measured in this study, training load and POMS fatigue levels increased significantly from February to March, likely reflecting an increase in the physical load associated with the start of team training following the off-season. Although the K6 scale scores showed an upward trend, they did not reach statistical significance. These results suggest that although players experienced physical fatigue due to increased training intensity, the notable adverse effects on their mental health were limited.

The robust regression analysis revealed a positive association trend between training load change and HOC change (*β* = 0.0003, *p* = 0.068). Animal and human studies have shown that exercise increases oxytocin levels (41), suggesting that the results of this study indicate that increases in physical load may be associated with accumulation of hair oxytocin.

In the change value analysis, a significant negative correlation was found between HCC change and POMS vigor change (rs = −0.44, *p* = 0.039). This finding suggests that decreases in HCC are associated with increases in vigor. A previous study (20) reported a negative association between HCC and vigor through cross-sectional analysis; however, this association was not confirmed in female athletes. The present study is important in that it demonstrates an association between changes in HCC and vigor in a longitudinal study of female athletes. Compared to cross-sectional studies, longitudinal analyses using change scores can account for temporal sequences, thus providing stronger evidence for causal inference. Therefore, the results of this study suggest that decreases in HCC may contribute to improvements in vigor in female soccer players, extending the findings of previous research.

The most notable finding of this study was the significant positive correlation between HOC change and K6 scale change (rs = 0.43, *p* = 0.043). This finding suggests that chronic elevation of oxytocin levels is associated with increased psychological stress. Oxytocin has been reported to increase recall of negative events and stressful stimuli (42, 43). Mechanistically, oxytocin may enhance emotional salience and increase attention to social and environmental cues, which under chronic stress conditions could amplify negative emotional processing rather than buffering against stress. Furthermore, prolonged oxytocin elevation may reflect sustained activation of stress-responsive neural circuits without achieving effective downregulation of the HPA axis, potentially indicating a dysregulated stress response system. These findings suggest that oxytocin may promote anxiety and fear in a context-dependent manner, and chronic elevation of oxytocin levels may play a different role from the anxiolytic effects observed with acute administration (29). That is, while acute increases in oxytocin may be effective in reducing stress, chronic elevation may reflect a compensatory response that fails to adequately regulate stress.

Importantly, the independence of HCC and HOC observed in this study warrants particular attention. Unlike the positive correlation reported in animal studies (30), we found no significant association between these hormones at either time point or in their changes. This discrepancy may reflect species differences, the specific characteristics of competitive athletes, or the distinct nature of stressors experienced by this population. This independence has practical implications: it suggests that relying on a single hormone biomarker would provide an incomplete assessment of an athlete's stress state. The fact that HCC changes were associated with vigor while HOC changes were associated with psychological distress further supports the notion that these hormones capture complementary rather than redundant information about stress and well-being.

While animal studies have reported a positive correlation between hair oxytocin and cortisol (30), this study found no significant correlations between HCC and HOC in February (rs = −0.03, *p* = 0.900), March (rs = −0.24, *p* = 0.283), or in their changes (rs = 0.28, *p* = 0.213). This discrepancy may be due to species differences, measurement subjects (animals vs. humans), or the characteristics of the specific population of competitive athletes. The results of this study suggest that these two hormones may fluctuate independently in female athletes. Furthermore, in this study, decreases in HCC were associated with increases in vigor, while increases in HOC were associated with increases in psychological stress (K6 scale scores). This finding is preliminary and requires cautious interpretation due to the limited sample size and measurement time points. However, it is possible that cortisol and oxytocin may be involved in different aspects of psychological states. Future research should examine in more detail the relationships between these two hormones and mental health using larger samples and multiple measurement time points.

The utility of combined HCC and HOC measurement is supported by three key findings from this study. First, changes in HCC were significantly associated with changes in vigor (a positive psychological state), while changes in HOC were significantly associated with changes in psychological distress (K6 scale), indicating that each hormone captures different aspects of psychological well-being. Second, the lack of correlation between HCC and HOC suggests that these hormones respond to stress through independent mechanisms, meaning that reliance on a single biomarker would provide an incomplete picture of the athlete's stress state. Third, while neither hormone showed significant mean-level changes despite increased training load and fatigue, the individual differences in hormone changes were meaningfully related to psychological outcomes, highlighting the value of examining within-person variability in stress biomarkers. Together, these findings suggest that the combined assessment of HCC and HOC provides complementary information that neither measure alone could capture.

### 4.1 Limitations

This study has some limitations. First, we did not measure cortisol in blood or saliva simultaneously with hair sampling, making it impossible to examine the relationship between transient cortisol and oxytocin levels and HCC and HOC effects. Previous research reported no correlation between HCC and blood cortisol concentration (20). Additionally, we confirmed that salivary cortisol and oxytocin levels at the time of measurement did not correlate with HCC or HOC, indicating that they are independent indicators (preliminary experimental data). Therefore, the HCC and HOC measurements in this study were likely not influenced by transient stress at the time of measurement.

Although this study targeted all players of a single university's women's soccer team, the measurement period occurred when senior players had retired and before new students joined, resulting in a limited sample size. Furthermore, this study focused on players from a women's soccer team with relatively similar lifestyle habits and stressors; thus, it remains unclear whether these results can be generalized to other women's soccer teams or male players. Questions remain regarding sex-based differences in HCC (9, 18), and the impact of HCC on mental health has been reported to be stronger in women than in men (19). Future studies should conduct analyses with more participants and verification in male soccer players. This study lacks true baseline measurements during a complete rest period, as the February measurement occurred shortly after the off-season rather than during it. The absence of blood or saliva samples also prevents comparison between acute and chronic hormonal markers. Additionally, training load was assessed via self-report rather than objective measures (e.g., GPS tracking, heart rate monitoring), which may introduce recall bias. However, self-reported training load has been validated against heart rate-based measures in previous research (4), supporting its utility as a practical assessment tool. Future studies should incorporate objective training quantification and multiple baseline assessments to strengthen causal inferences. Menstrual cycle phase and hormonal contraceptive use were not recorded in this study. Given that both cortisol and oxytocin levels can be influenced by ovarian hormone fluctuations across the menstrual cycle, this represents a limitation that should be addressed in future research. Additionally, the internal consistency of the fatigue subscale was relatively low (Cronbach's *α* = 0.56–0.59), which may have attenuated the observed associations between hair hormones and fatigue. Finally, regarding the mechanism of interaction between oxytocin and cortisol, it is difficult to infer causal relationships from the observational design of this study. Future research should employ experimental approaches to elucidate these hormonal interactions in more detail, as well as approaches involving long-term measurements and analyses.

## 5 Conclusion

This study showed an association trend between increased training load and increased HOC, and between physical aspects stressor and increased HCC, suggesting that both training load and physical stress may be associated with the accumulation of hair oxytocin and hair cortisol, respectively. Additionally, while decreases in HCC were associated with increases in vigor, increases in HOC were associated with increases in psychological stress. Importantly, no significant correlation was found between HCC and HOC, suggesting that these two hormones fluctuate independently and may be involved in different aspects of psychological states.

These findings demonstrate the importance of measuring both cortisol and oxytocin in the assessment of chronic stress in female athletes. Combined measurement of HCC and HOC may contribute to a more comprehensive evaluation of the accumulation of physical stress from training and its psychological impacts. This measurement approach has the potential to function as a physiological indicator that visualizes the stress states of individual players and contributes to the early detection of burnout and overtraining, thereby providing a new perspective for athlete health management and performance enhancement.

For practitioners, we recommend incorporating periodic hair hormone assessments (e.g., monthly during intensive training periods) alongside psychological monitoring to identify athletes who may be experiencing chronic stress that is not fully captured by self-report measures alone. This dual biomarker approach may facilitate early intervention and individualized load management before the onset of overtraining syndrome or burnout.

## Data Availability

The raw data supporting the conclusions of this article will be made available by the authors, without undue reservation.
